# Coronal Microleakage for Readymade and Hand Mixed Temporary Filling Materials

**Published:** 2011-11-15

**Authors:** Hager Ibn Idriss Aledrissy, Neamat Hassan Abubakr, Nadia Ahmed Yahia, Yahia Eltayib Ibrahim

**Affiliations:** 1. Department of Conservative Dentistry, Dental School, EL Neelan University of Medical Sciences, Khartoum, Sudan.; 2. Department of Conservative Dentistry, Dental School, Khartoum University of Medical Sciences, Khartoum, Sudan.; 3. Department of Basic Sciences, Dental School, Khartoum University of Medical Sciences, Khartoum, Sudan.

**Keywords:** Coronal, Dental Leakage, Dental Restoration, Penetration, Temporary

## Abstract

**INTRODUCTION:**

The purpose of this in vitro study was to evaluate the sealing ability of the readymade temporary filling and hand mixed materials by assessing coronal microleakage.

**MATERIALS AND METHODS:**

Standardized access cavities were prepared in 80 intact human permanent premolar teeth. They were divided randomly into four experimental groups (n=20). The teeth were restored using one of the temporary materials including Cavisol, Litrak, Zinc phosphate cement, Zinconol (IRM). Thermocycling was applied on the specimens. Methylene blue dye was applied and penetration was evaluated under stereomicroscope. Grading of the microleakage pattern was from 1 to 3, with 3 providing the best seal. Results were analyzed using one-way ANOVA test (P<0.05).

**RESULTS:**

Microleakage of Cavisol and Litrak samples achieved grade 3; whereas zinc phosphate cement and Zinconol samples absorbed the dye into the bulk of the materials. Cavisol was found to exhibit the best seal amongst the four tested materials followed by Litrak, zinc phosphate cement, and Zinconol. There was a statistically significant difference in the microleakage scores obtained between the materials (P<0.01).

**CONCLUSION:**

Among the four materials tested, readymade temporary filling provided the best sealing ability over hand-mixed. This study emphasizes the importance of correct placement and sufficient thickness of temporary filling materials in endodontic access cavities to ensure a tight seal.

## INTRODUCTION

Loss of integrity of coronal tooth substances and invasion of microorganism into dentine and pulp space play an important role in pulpal and periradicular diseases. Inadequate coronal seal allow biological contamination and penetration of saliva, nutrients, chemicals and also microorganisms and their byproducts. Coronal microleakage appears to be of equal or greater clinical relevance as a factor in endodontic failure than apical leakage due to risk of recontamination [1][2][3][4][5]. A recent study concluded that food-derived microbiota could enter the necrotic root canal system via coronal microleakage [6].

A coronal filling material is considered effective when it is able to fulfill certain properties including an effective seal of tooth margins, lack of porosity and dimensional stability to thermal changes, good abrasion and compression resistance, ease of insertion and removal, compatibility with intra-canal medicament and good aesthetic appearance [7][8][9].

Several studies evaluating the microleakage of temporary restorative materials have been conducted and the used techniques were mostly assessed seal ability using dye penetration with either thermal cycling or load cycling procedures [7][9][10][11][12][13]. Cruz et al. concluded that thermal cycling procedures seemed to affect the sealing ability of certain types of temporary endodontic filling materials whilst load cycling did not [14].

Most of the conducted studies focused on the sealing ability of Zinconol (IRM) which exhibited gross microleakage [15]; Zmener et al. in a dye penetration study found that IRM specimens absorbed the dye into the bulk of the material [16].

The aim of this study was to evaluate the sealing ability of readymade and hand mixed temporary filling materials by conducting microleakage tests using methylene blue dye.

## MATERIALS AND METHODS

Eighty caries free extracted human maxillary and mandibular premolars that had been stored in 10% formalin were cleaned of soft tissue and debris, rinsed overnight in running water and then immersed in deionized water for 24 hours.

**Figure 1:**
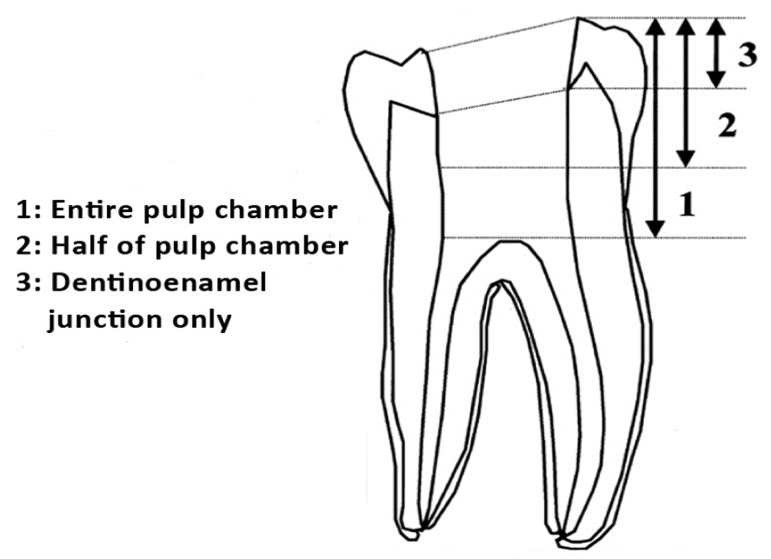
Grades of dye penetration

Standardized coronal access cavities to the pulp chamber were prepared in the occlusal surfaces with the aid of a template measuring 4mm×4mm; access was made using a high speed air turbine under water coolant with a round bur for initial entry and a diamond fissure bur to extend the preparation to the desire occlusal outline. All teeth were irrigated using 5% sodium hypochlorite (Hyposol, Prevest Denpro Ltd, India) to remove remaining smear layer, pulp tissues and other debris inside the pulp chamber. The prepared openings were air dried and cotton pellet were placed on the floor of the pulp chamber. A periodontal probe was used for measuring the final depth cavity and assuring that it could accommodate at least 4mm of the temporary filling material. The teeth were divided randomly into four groups of 20 teeth each (Table 1). All materials were mixed and handled according to the manufactures’ instructions. The filling materials were incrementally introduced into the access opening from the bottom up with the use of a plastic instrument every effort was made to ensure that the filling materials were carefully pressed against the cavity walls. The specimens were then placed in normal saline and stored in an incubator (GallenKamp, London, UK) at 37ºC for 48 hours to ensure setting of the materials. All experimental groups underwent thermocycling for 500 cycles in distilled water at 5ºC and 55ºC with a dwell time of 30 seconds in each bath using thermocycling machine (Techne, Staffordshire, UK). After thermal shocks, all specimens were placed in normal saline and incubated for 24 hours for at 37ºC for complete setting. The specimens were dried and painted with two layers of nail varnish except 1mm around the restoration margin to prepare them for leakage assessment. All specimens were allowed to dry for one hour and then coated with inlay wax twice before been placed in 2% methylene blue dye solution (pH=7.4). Specimens were kept for 10 days at 37ºC in the incubator. They were then washed under running water, dried, and longitudinally sectioned in the a mesiodistal direction using a low speed diamond blade under constant water lubrication to remove the debris and smear layer created by cutting. Sections were stored at 37ºC in an incubator. Dye penetrations were measured in millimeters, using calibrated stereomicroscope (WILD Heerbrugg wild, Switzerland) at ×4 magnification. The measurement of dye penetration was jointly carried out by two researchers using a modification of scoring technique introduced by Lee et al. (Figure 1) [12]. Data was analyzed using one way ANOVA test (P<0.05) to determine if a statistically significant difference existed between the groups of the tested materials.

**Table 1:** Investigated materials

**Material**	**Composition**	**Batch No.**	**Manufacturer**
**Cavisol**	Zinc oxide, Calcium sulfate, Zinc sulfate, Plasticizers, Resins, Pepper-Mint aroma and Excipients	1707	Golchai Co. Iran
**Litrak**	Zinc oxide, Zinc sulfate, Calcium Sulfate, Plasticizers, Resins, Mint aroma and Excipients	0457340113	LASCOD Sesto, Via L. Longo, Florence, Italy
**Zinconol**	*Powder:* Zinc oxide and Polymer *liquid:* Eugenol and Resin	89011	Prevest Denpro Lt, Jammu 180010 India
**Zinc Phosphate Cement**	*Powder:* Zinc phosphate, Magnesium oxide *Liquid:* Phosphoric acid	0310009	Scitem Limited, London SW 19, 5HP, UK

## RESULTS

All samples were screened visually and then under the stereomicroscope. Amongst four tested materials, Cavisol showed the least microleakage, followed by Litrak, zinc phosphate and Zinconol. The results showed there were statistically significant differences between tested materials. A Kruskal Wallis (ANOVA) test revealed a significant difference between four materials (P<0.01).

Post hoc test using Mann-Whitney U tests revealed a significant difference between the readymade and hand mixed one (P<0.01).

**Figure 2:**
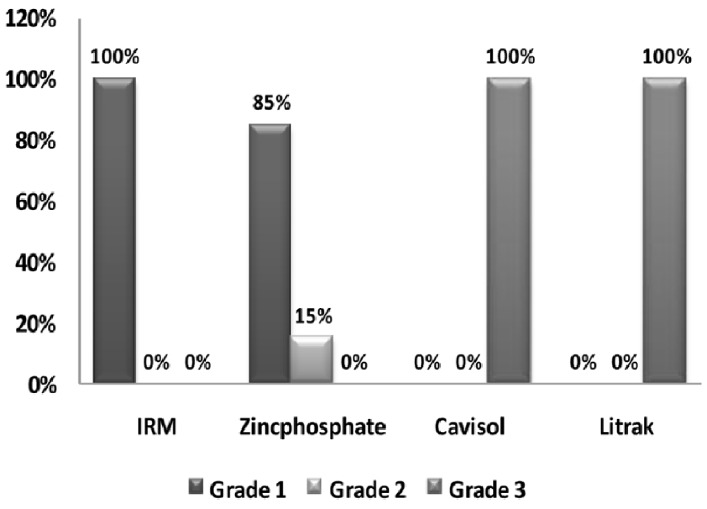
Relation between the microleakage grade and different materials

**Figure 3:**
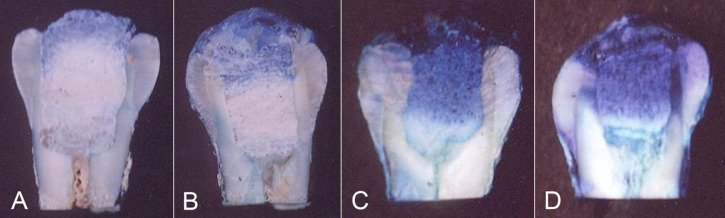
Micrograph of dye penetration within the examined materials A: Cavisol exhibited grade 3. B: Litark exhibited grade 3. C: Zinc phosphate exhibited grade 1. D: Zinconol exhibited grade 1.

Cavisol and Litrak samples showed the least microleakage (leakage grade 3; 100%), followed by zinc phosphate cement which had leakage grade 1 (85%) and grade 2 (15%).Zinconol samples had the worse results with 100% of samples scoring grade 1 (Figure 2).Micrograph shows the leakage of the tested materials. Dye penetration into the material was noted in Cavisol (Figure 3A), Litrak (Figure 3B), zinc phosphate (Figure 3C), all zinconol specimens exhibited total leakage absorbed the dye to the bulk of the material (Figure 3D).

## DISCUSSION

In this in vitro study, readymade filling materials (Cavisol and Litrak ) were compared to hand mixed (zinconol and zinc phosphate) temporary restorative materials. The experiment was conducted on extracted intact premolars with 4mm thickness of temporary restorative materials conforming to previous reports [17][18][19], which advised a minimum of 3.5 to 4mm of restorative material to prevent microleakage. However, clinically a 4 to 5mm thickness of temporary restorative material cannot always be achieved, in particular not in severely broken-down teeth requiring endodontic therapy. Access preparations can frequently be made in premolars with minor loss of coronal structure or with existing four-surface class 1 or three-surface class 2 restorations.

To prevent weakening of the tooth, coronal structure should be preserved whenever possible [9], whereas temporary restorative materials need adequate retention to prevent dislodgement between appointments. Therefore, a 4mm thick temporary restorative material is desirable. Often, teeth that require endodontic therapy have lost so much tooth structure that less than minimum recommended thickness of access preparation is sometimes available. A number of methods have been used to evaluate the microleakage of temporary endodontic filling materials [10][16][20][21]. Dye penetration is generally recognized as an good indicator of bacterial invasion. Therefore, bacterial invasion to root canal may occur when dye penetration depth is greater than the thickness of temporary restorative material. In such a circumstance, the temporary restorative material provides no protection against bacterial infection [5].

The present study utilized thermal cycling procedure to simulate intraoral conditions. The temperature range of 55±2ºC and 5±2ºC used in this study corresponds to the extremes of temperatures that could be experienced in the oral environment [10][18][19]. The rationale for selecting a 10 day observation period was based on the premise that this is an adequate time-lapse for a temporary restoration between endodontic appointments, at which no or minimum leakage can occur.

Our observations revealed that all tested materials leaked to some extent. Different authors have reported conflicting results concerning the ability of readymade temporary filling materials such as Cavit and hand mixed materials to prevent coronal microleakage. Some revealed that Cavit a readymade material had the best sealing ability whereas IRM as hand mixed showed the maximum dye penetration [15]. Other indicated that Cavit showed less microleakage in dye penetration [22], and act as a barrier to leakage than IRM [23]. An additional study indicated that IRM specimens absorbed the dye into the bulk of the materials [16]. A similar finding was noted in this study. This finding could probably be attributed to the instability of zinc oxide when subjected to extreme of temperatures [20], as well as inconsistencies in the mixing process and the resulting lack of homogeneity [24]. Cavisol and Litrak are a premixed, ready to use, hygroscopic material that expand when in contact with moisture, and presumably this expansion permits the material to adapt more tightly to dentin walls, thus providing a good seal under different conditions, including Thermocycling. In an in vitro study Jenkins et al. indicated that Tetric (bonded composite) showed a better sealing ability than Cavit and Pro Root MTA [25]. Thus, the added benefit of an orifice barrier to reduce coronal leakage may help in retaining endodontically treated teeth.

## CONCLUSION

Readymade temporary filling had superior sealing ability over hand-mixed. This emphasize the importance of material and correctly placing a sufficient thickness of temporary filling materials in endodontic access cavities to ensure a tight seal. However, further research such as a long-term study may provide better evidence.
